# Control of Precursor Maturation and Disposal Is an Early Regulative Mechanism in the Normal Insulin Production of Pancreatic β-Cells

**DOI:** 10.1371/journal.pone.0019446

**Published:** 2011-04-29

**Authors:** Jie Wang, Ying Chen, Qingxin Yuan, Wei Tang, Xiaoping Zhang, Kwame Osei

**Affiliations:** 1 Department of Internal Medicine, The Ohio State University, Columbus, Ohio, United States of America; 2 Departments of Neurobiology and Neurology, The University of Chicago, Chicago, Illinois, United States of America; University of Tor Vergata, Italy

## Abstract

The essential folding and maturation process of proinsulin in β-cells is largely uncharacterized. To analyze this process, we improved approaches to immunoblotting, metabolic labeling, and data analysis used to determine the proportion of monomers and non-monomers and changes in composition of proinsulin in cells. We found the natural occurrence of a large proportion of proinsulin in various non-monomer states, i.e., aggregates, in normal mouse and human β-cells and a striking increase in the proportion of proinsulin non-monomers in *Ins2^+/Akita^* mice in response to a mutation (C96Y) in the insulin 2 (*Ins2*) gene. Proinsulin emerges in monomer and abundant dual-fate non-monomer states during nascent protein synthesis and shows heavy and preferential ATP/redox-sensitive disposal among secretory proteins during early post-translational processes. These findings support the preservation of proinsulin's aggregation-prone nature and low relative folding rate that permits the plentiful production of non-monomer forms with incomplete folding. Thus, in normal mouse/human β-cells, proinsulin's integrated maturation and degradation processes maintain a balance of natively and non-natively folded states, i.e., proinsulin homeostasis (PIHO). Further analysis discovered the high susceptibility of PIHO to cellular energy and calcium changes, endoplasmic reticulum (ER) and reductive/oxidative stress, and insults by thiol reagent and cytokine. These results expose a direct correlation between various extra-/intracellular influences and (a)typical integrations of proinsulin maturation and disposal processes. Overall, our findings demonstrated that the control of precursor maturation and disposal acts as an early regulative mechanism in normal insulin production, and its disorder is crucially linked to β-cell failure and diabetes pathogenesis.

## Introduction

Pancreatic β-cell failure, which is central in the pathogenesis of diabetes, has been explained by autoimmune assault in type 1 diabetes (T1D) [1] and by glucolipotoxicity, amyloid deposition, insulin resistance, unfolded protein response (UPR), and endoplasmic reticular (ER) and/or oxidative stress in type 2 diabetes (T2D) [2]–[14]. However, it remains unclear whether there is an intrinsic process that is susceptible to these diabetes-related influences and linked as well to β-cell dysfunction in all forms of diabetes. Among monogenic disorders, early studies characterized syndromes similar to T2D that were caused by mutations in the insulin (*INS*) gene in non-neonatal patients [15]. In Akita mice, we identified a mutation (*Ins2^+/Akita^*, C96Y) that did not remarkably attenuate synthesis (at early ages) but that did cause abnormal aggregation and degradation of cellular (pro)insulin [16], which, in turn, produced numerous intracellular toxic consequences, abnormalities in insulin secretion, β-cell depletion, and early onset diabetes [16]–[18]. Similar pathogenesis is implicated in up to 20% of neonatal diabetes that associates with defects in the *INS* gene and with proband variants for T1D, maturity onset diabetes of the young, and even T2D [19], [20]. Those subjects with defects in the same preproinsulin molecule showed similar β-cell failure as that seen in general diabetes, such as hyper- or hypo(pro)insulinemia. Therefore, we suggest that the misfolding and impaired maturation of proinsulin is linked to β-cell failure in all diabetics.

Proinsulin is the dominant form of insulin in the early secretory pathway following the rapid removal of signal peptide from preproinsulin and conversion of the natively folded proinsulin into mature insulin [15]. Insulin is the most abundant protein product of β-cells and constitutes up to 14% of the dry weight of rodent islets/β-cells [21], [22]. Studies of protein biosynthesis in rodent/carp islets have shown incorporation of 6 to 30% of radioactive amino acids into preproinsulin in basal or glucose-stimulated conditions [23], [24], although islets/β-cells produce more than 20,000 proteins. Thus, proinsulin bears the greatest burden in β-cell protein folding. Since the discovery of proinsulin in 1967 [25], metabolic-labeling studies have consistently demonstrated the rapid appearance of monomers in the ER supports the long-held belief that proinsulin rapidly achieves its native conformation [15]. As well, Huang and Arvan [26] have indicated that intracellular disulfide bonds form quickly in most proinsulin monomers and exit the ER in about 20 minutes.

However, the folding rate of cellular proinsulin has not been determined. In this study, we define a relative folding rate as the percentage of natively folded proinsulin monomers in a given period in all synthesized proinsulin polypeptides. These polypeptides include proinsulin monomers and all other non-natively (incompletely/incorrectly) folded proinsulin aggregates other than monomer states (non-monomers). With a rate of proinsulin folding well below 100%, the natural instability of the non-natively folded polypeptides would contribute to the appearance of a fraction of non-natively folded proinsulin in β-cells with possible aggregation for further maturation or clearance. We previously noted remarkable proinsulin aggregation in normal islets in Akita mice with significantly increased proinsulin aggregates resulting from an *Ins2^+/Akita^* mutation. However, we chose not to examine proinsulin aggregation in normal β-cells in that study [16], [17], partly because the states of endogenous proinsulin have not been demonstrated despite satisfactory documentation of labeled nascent proinsulin monomers by many pulse-chase studies and because no well developed immunoblotting approaches have been employed in (pro)insulin study, though such approaches have been applied extensively in determining numerous protein states/levels. Improved approaches are required for better visualization of the states/bands of immunoreactive (pro)insulin in whole-cell protein pools in the study of β-cell biology. Moreover, despite much progress in biomedical studies, methods for analyzing protein folding *in vivo* remain suboptimal [27] and prevent our discerning the entire mechanism of proinsulin maturation and its link with β-cell (dys)function.

We have used our improved approaches to metabolic labeling and C-peptide immunoblotting to visualize proinsulin aggregates in normal islets [16], [17] and applied them in this study to determine the natural occurrence of a significant proportion of proinsulin in normal β-cells as various non-monomer states. We could ascertain that the aggregation-prone nature and very low relative folding rate of proinsulin maintains a balance of non-natively and natively folded states through the integration of maturation and degradation processes in normal β-cells and that PIHO is highly susceptible to various extra-/intracellular influences. Our findings demonstrated that the process of proinsulin maturation determines the rate and efficiency of insulin biosynthesis and led us to hypothesize a model of PIHO in β-cell (dys)function.

## Results

### A significant fraction of proinsulin exists in non-monomer states in the whole-cell protein pool of β-cells in normal mice

**Figure 1:**
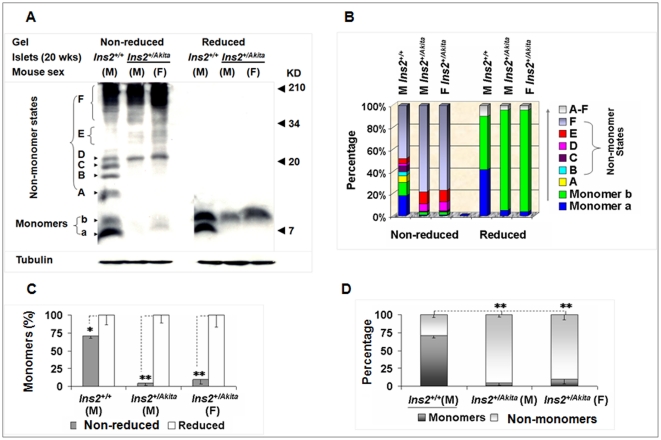
A significant fraction of proinsulin exists in non-monomer states in the whole-cell protein pool of normal mouse islets. (A). Equal amounts (30 µg) of *Ins2^+/+^* and *Ins2^+/Akita^* islet proteins in the RIPA buffer were resolved by reduced (100 mM DTT) and non-reduced tricine-SDS-PAGE (16.5%T, 6% C; without urea) and then subjected to C-peptide immunoblot analysis. M, male; F, female. (B) Percentage of each state in (A). (C) The relative levels of proinsulin monomers under the 2 conditions in (A). (D) The proportions of proinsulin monomers and non-monomers in the normal and mutant islets in (A) that were calculated by using the method introduced in “Materials and Methods.” The data in (C) and (D) were reported as mean ± SD. *P<0.05; **P<0.005.

**Figure 2:**
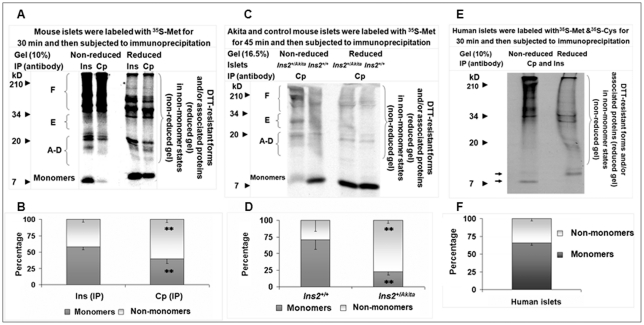
A significant proportion of nascent proinsulin emerges in non-monomer states during nascent protein synthesis in mouse and human islets. Labeled mouse (A and C) or human (E) islet proteins were subjected to IP as indicated in A, C, and E. Equal amounts of individual immunoprecipitates were resolved by non-reduced and reduced tricine-SDS-PAGE for autoradiography. Proinsulin monomer and non-monomer proportions in individual immunoprecipitates in A, C, and E that were calculated using the method introduced in “Materials and Methods” were shown in B (A), D (C), and F (E). Note the image in (C) is the radioautograph of a membrane prepared by the same procedure for immunoblotting (Fig. 1A), and the slightly unusual position of proinsulin monomers at reduced versus non-reduced condition in (C) resulted from a margin effect of electrophoresis. In addition, adherence of small polypeptides to the membrane in (C) at protein transfer reduced the transfer efficiency of large proteins. As a consequence, the signal of proinsulin non-monomer states under non-reduced condition or of unidentified molecular helpers (e.g., possible protein disulfide isomerases) under reduced condition (apparent in the upper area of the membrane) was weaker than the original radioactivity (Fig. 2A, C). Arrows in (F), proinsulin monomers; Cp, C-peptide; Ins, insulin; IP, immunoprecipitation. Data in (B), (D), and (E) were reported as mean ± SD. **P<0.005.

**Figure 3:**
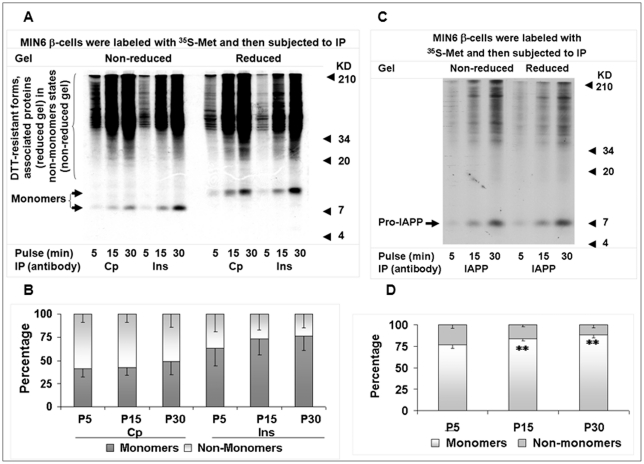
Proinsulin maintains a significant proportion of non-monomer states in the homeostasis of secretory proteins in an extended *de novo* synthesis course. MIN6 β-cells were labeled for 5, 15, and 30 minutes at the 25.5 mM glucose condition, and cellular proteins were then subjected to serial IP with insulin (Ins), C-peptide (Cp), or IAPP antisera. Equal amounts of Ins, Cp, or IAPP immunoprecipitates were resolved by 10% non-reduced and reduced tricine-SDS-PAGE for autoradiography. The monomer and non-monomer proportions of proinsulin in Ins or Cp immunoprecipitates (A) or pro-IAPP in IAPP immunoprecipitates (C) that were calculated using the method introduced in “Materials and Methods” were shown in B (A) and D (C). P5, 5-min pulse; P15, 15-min pulse; P30; 30-min pulse. Data in (B) and (D) were reported as mean ± SD. **P<0.005.

**Figure 4:**
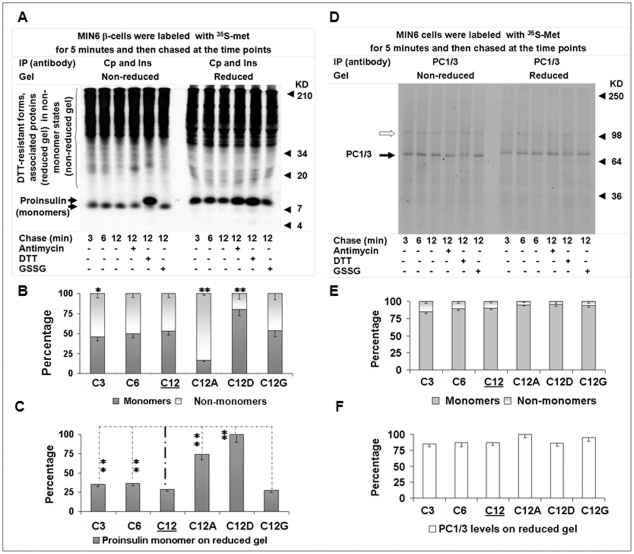
Nascent proinsulin kinetics in states and levels during early post-translational processing. MIN6 β-cells were chased for the indicated times with/without antimycin (10 uM), DTT (5 mM), or GSSG (50 uM) after a 5-min pulse, and cellular proteins were then subjected to serial IP with insulin (Ins) and C-peptide (Cp) antisera or PC1/3 antisera. Equal amounts of Ins and Cp immunoprecipitates (A) or PC1/3 immunoprecipitates (D) were respectively resolved by 10% tricine or 7.5% Laemmli non-reduced and reduced SDS-PAGE for autoradiography. The monomer and non-monomer proportions of proinsulin or PC1/3 in individual immunoprecipitates that were calculated using the method introduced in “Materials and Methods” were shown in B (A) and E (D). Relative levels of nascent proinsulin monomers in (A) or PC1/3 monomers in (D) of individual immunoprecipitates (on the reduced gel) were shown in C (A) and F (D). Empty arrow in (D), possible PC1/3 precursor. Data in (B), (C), (E), and (F) were reported as mean ± SD. *P<0.05; **P<0.005.

**Figure 5:**
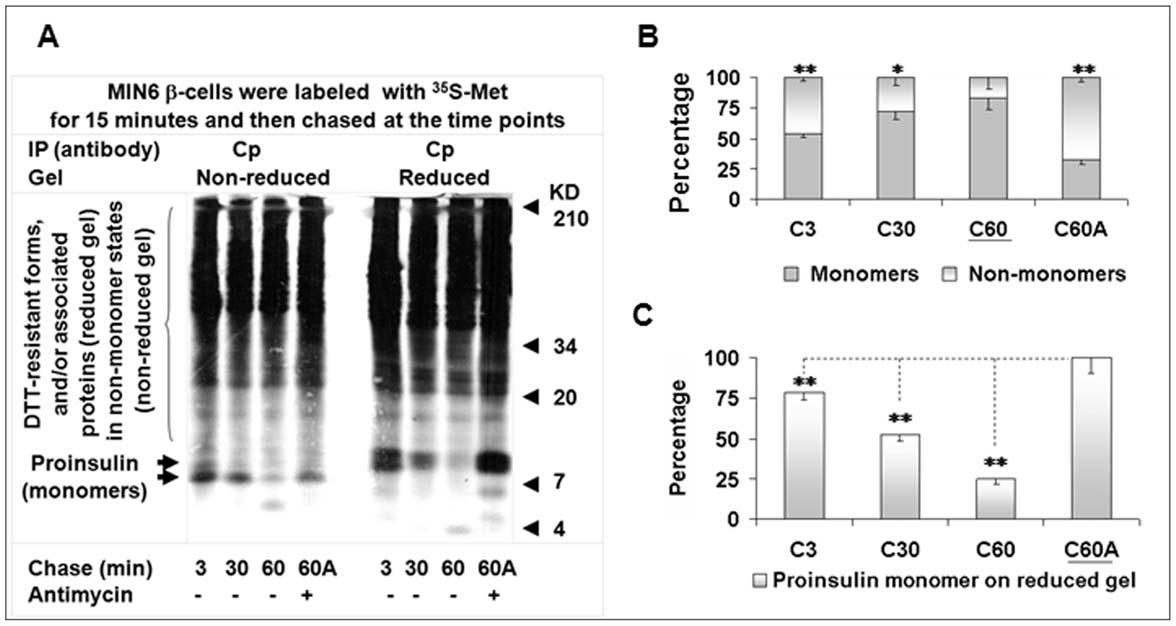
Prolonged residual non-monomer fraction and rapid disposal of nascent proinsulin in the post-translational process. MIN6 β-cells were chased for the indicated times with/without antimycin (10 uM) after a 15-min pulse, and cellular proteins were then subjected to IP with C-peptide (Cp) antisera. Equal amounts of individual Cp immunoprecipitates were resolved by 10% non-reduced and reduced tricine-SDS-PAGE for autoradiography (A). Graph (B) showed the proportions of proinsulin monomers and non-monomers in individual Cp immunoprecipitates that were calculated using the method introduced in “Materials and Methods.” Graph C showed the relative levels of nascent proinsulin monomers (on reduced gel) in individual Cp immunoprecipitates in (A). Data in (B) and (C) were reported as mean ± SD. *P<0.05; **P<0.005.

**Figure 6:**
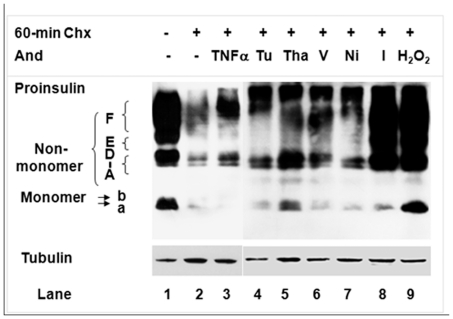
Effects of various reagents on proinsulin states during a 60-min post-translational process. We incubated MIN6 β-cells in culture medium with/without the following reagents for 60 minutes: cycloheximide (Chx; 100 µg/mL); Chx and TNFα (2 ng/mL); tunicamycin (Tu, 1 ug/mL); thapsigargin (Tha, 2 uM); verapamil (V, 100 uM); nickel chloride (Ni, 1 mM); iodoacetamide (I, 1 mM); or H_2_O_2_ (250 uM). Cellular proteins were extracted in the tricine sample buffer (Bio-Rad) by SPP-B, resolved by tricine-SDS-PAGE without urea (16.5%T, 5% C), and then subjected to C-peptide and tubulin immunoblot analysis. Protein, 30 µg per lane.

For immunoblot analysis, we extracted whole-cell proteins from mouse islets in a radioimmunoprecipitation assay (RIPA) buffer [16] using sample preparation procedure A (SPP-A) (detailed in “Materials and Methods”). In collecting protein samples in all studies described, we included 20 mM of N-ethylmaleimide to block free thiols. C-peptide immunoblot analysis detected various non-monomer and 2-monomer states of proinsulin under non-reduced condition in normal *Ins2^+/+^* islets (Fig. 1A; Figure S1A shows their molecular weights calculated). Consistent with our previous work [16], we added dithiothreitol (DTT) to create a reduced condition and thereby observed significant disappearance of the signal of non-monomer states with simultaneous enhancement of the signal of proinsulin monomers (Fig. 1A). However, we observed some DTT-resistant signals in non-monomer areas in images with long exposures (data not shown). We considered that monomer *a* likely represented the proinsulin with native conformation because of its general preponderance in normal islets (Fig. 1A) and cloned mouse β-cells (Fig. S1B) and its migration pattern that resembled that of human proinsulin markers (unpublished observation). As observed in metabolic-labeling studies, form *b* may be an isomer [28] because its abundance was secondary in normal islets but richer in the *Ins2^+/Akita^* islets under non-reduced condition. Form *b* may also be a mixture of the same isomers observed on high concentration tricine sodium dodecyl sulfate polyacrylamide gel electrophoresis (SDS-PAGE) with urea [28] (unpublished observation); resolution of the applied tricine SDS-PAGE without urea was limited. Variation in control of tubulin was not as great as that of proinsulin. Consistent with previous observations [16], [26], proinsulin monomers also migrated slightly slower on the tricine gel under reduced than non-reduced condition in most cases (Figs. 1, 2, 3, 4, 5, 6).

Quantitative analysis (detailed in “Materials and Methods”) revealed that the immunoreactivity of the non-monomer states represented most of the total C-peptide immunoreactivity in the mutant islets, and represented a significant fraction of the total C-peptide immunoreactivity in normal islets under non-reduced condition (Figs. 1A–1D; Tables S1, S2, S3). Among the diverse non-monomer states, those in Areas A to C were richer in normal islets, but those with high molecular weights in Areas D to F were more abundant as severe aggregates in the mutant, regardless of their currently unknown natures (Fig. 1A, B). The much higher level of proinsulin in normal islets than in the mutant (Fig. 1A) precludes a causal link in the whole-cell protein pools between the level of proinsulin and the proportion of proinsulin non-monomers. Together, the above observations suggest a disturbance in the normal balance of proinsulin monomer and abundant non-monomer states in the mutant islets.

We also determined whether the pattern of proinsulin states can be preserved in early cloned MIN6 β-cells and recently established *Ins2^+/+^* and *Ins2^+/Akita^* β-cells [18]. C-peptide immunoblot analysis showed a similar pattern of proinsulin states in MIN6 and *Ins2^+/+^* β-cells to that in normal islets, whereas the pattern in *Ins2^+/Akita^* β-cells resembled that in mutant islets (Fig. 1A, S1B). Differences in the applied gels and protein extract buffers (detailed in “Materials and Methods”) as well as possible variations between islets and cloned β-cells could produce slight discrepancies in migration or density of some states. Similarly, monomer *b* detected in immunoblotting (Fig. 1A, 16.5% tricine gel) did not appear in most autoradiographs (Figs. 2A, 3A, 4A, 10% tricine gel), though it was visible in the image with longer electrophoresis (Fig. 5A).

### A significant fraction of proinsulin emerges in non-monomer states during nascent protein synthesis in normal mouse/human β-cells

To investigate the basis for the occurrence of proinsulin non-monomer states, we used basically the same labeling and immunoprecipitation (IP) method applied by Steiner's group [29] and examined whether non-monomers appear quickly during *de novo* proinsulin synthesis. The radioautograph shows approximately 58% monomers and 42% non-monomers precipitated by insulin IP and about 40% monomers and 60% non-monomers recovered by C-peptide IP from islet proteins labeled 30 minutes with ^35^S-methionine (Met) (Fig. 2A, B; Table S4). (Applying ^35^S-Met simplifies the experiment because its residue occurs in the dominant proinsulin 2 form and not in proinsulin 1 in mice). The data indicate that nascent proinsulin non-monomers shape quickly and have lower affinity with the high conformation-dependent insulin antisera [30] than with C-peptide antisera. Therefore, recovery of the proinsulin non-monomers by IP using only insulin antisera is relatively limited because of the well known high dependence of insulin antisera on conformation, which requires the natively folded proinsulin to generate specific recognition epitope(s) [30]. On the other hand, C-peptide antisera react with an unstructured peptide whether free or within the proinsulin molecule. These properties enhance detection of non-natively folded proinsulin molecules by C-peptide antisera regardless of their state in processes. We also found that approximately 34% nascent proinsulin non-monomers appeared in 30-minute labeled human islets (Fig. 2E, F; Table S6), and that the completely folded ^125^I-proinsulin marker (Linco) in islet protein extracts that was subjected to the same IP and electrophoresis procedure did not form significant aggregates (Fig. S2, Table S7). Moreover, the proinsulin states of *Ins2^+/Akita^* islets (Fig. 1) were similarly disturbed during nascent protein synthesis (Fig. 2C, D; Table S5). These data suggest that the proinsulin non-monomer states of islets/β-cells are primarily shaped *in vivo* prior to protein extractions and result mainly from non-natively folded proinsulin.

### Proinsulin maintains a significant proportion of non-monomer states in the homeostasis of secretory proteins in an extended *de novo* synthesis course

Because islets of various sizes have different β-cell numbers and MIN6 β-cells are known to preserve the basic characteristics of their pancreatic β-cell progenitors, we further investigated the basic properties of endogenous proinsulin states in MIN6 β-cells. We found a gradual increase in the proportion of islet amyloid polypeptide precursor (pro-IAPP) monomers from 77 to 88% from 5 to 30 minutes during a pulse course. Strikingly, the proportion of ^35^S-proinsulin monomers increased from 41 to 49% in C-peptide immunoprecipitates and from 64 to 76% in insulin immunoprecipitates (Fig. 3A–D; Tables S8, S9). Just as in whole-cell protein pools (Fig. 1), we found no causal link between the level of proinsulin and the proportion of proinsulin non-monomers in nascent protein pools; we did observe an anti-parallel relationship between the ^35^S-proinsulin/^35^S-pro-IAPP level and non-monomer proportion during this synthesis course. Prohormone convertase 1/3 (PC1/3), another internal control, reached up to 85% in a 3-minute chase after a 5-minute pulse (Fig. 4D, E). These data indicate that proinsulin maintains most abundant non-monomer states among these nascent secretory proteins, though all these proteins must form intramolecular disulfide bonds in the ER and are targeted for insulin granules following synthesis.

In general, the structures of protein non-monomers are non-natively folded, and those of monomers are natively folded [27], [31] irrespective of inclusion of possible isomers. Therefore, the high percentage of proinsulin non-monomers that constantly appeared among normal islet/β-cell proteins (Figs. 1, 2, 3, 4, 5, 6) indicates a low relative folding rate of proinsulin. Moreover, comparison of about 10 islet proteins (Fig. S3) demonstrates that the folding rate of proinsulin is the lowest known.

### Heavy and preferential ATP/redox-sensitive disposal and non-monomer dual fate of nascent proinsulin during the early post-translational process

After a 5-minute pulse, MIN6 β-cells were chased at 3, 6, 12, and 12 minutes with antimycin (10 µM), DTT (5 mM), or oxidized glutathione (GSSG; 50 µM). From the 3- to 12-minute natural chase, the fraction of proinsulin monomers increased steadily from 46 to 53%, or the fraction of control PC1/3 monomers increased from 85 to 90% (Fig. 4A, B, D, E; Tables S10, S11). Folding efficiency increased slightly from the 3- to 12-minute chase by removal of more non-monomers than monomers, and as the monomer and non-monomer proportions fluctuated, the level of ^35^S-proinsulin decreased 7% (Fig. 4A, C; Table S12). The decrease was not due to conversion of ^35^S-proinsulin into insulin because this customary route for nascent proinsulin is inactive during this limited chase course [15], [26]. Among the various 12-minute chases, the monomer fractions of nascent PC1/3 changed slightly (<15%) (Fig. 4D, E; Table S11), but those of proinsulin changed radically. Compared to about 53% at the 12-minute natural chase or with addition of GSSG, the fraction decreased to 17% with antimycin treatment, which resulted from the blocking of the *in vivo* conversion of proinsulin non-monomers to monomers and from the blocking of the heavy removal of proinsulin non-monomers (Fig. 4A, B; Table S10). In contrast, adding DTT during the 12-minute chase increased the ^35^S-proinsulin monomer fraction to 80%, mainly from the *in vivo* conversion of most non-monomers to monomers.

Unexpectedly, comparisons of the natural 3- to 12-minute chases to the 12-minute chases with addition of antimycin or DTT disclosed an additional bulk removal of ^35^S-proinsulin in the MIN6 cells. The level of ^35^S-proinsulin plateaued with DTT treatment rather than in the expected 3-minute chase, which was the shortest chase after the 5-minute pulse (Fig. 4A, C; Table S12). Compared to the plateau (100%), around 74% of proinsulin was retained with antimycin treatment, 35 to 36% in the 3- or 6-minute chase, and 27 to 29% in the 12-minute chase with or without GSSG. These data indicate that over 60% of ^35^S-proinsulin was removed within a limited time in the post-translational processing. In contrast, PC1/3 levels varied less than 15% among the diverse chases (Fig. 4D, F; Table S13). A latent but powerful substrate-selective disposal process, termed “rapid disposal” (RD), has thus been found in the early post-translational processing of proinsulin.

Addition of DTT reduced the disulfide bonds of proinsulin *in vivo* in a process reflected by a slightly slower migration of proinsulin monomers on the gel (Fig. 4A), which consistently indicated that proinsulin *per se* is sensitive to DTT before it exits the ER [26]. Importantly, the addition of DTT also led to proinsulin non-monomer dissociation with unidentified DTT-sensitive molecular helpers (e.g., possible protein disulfide isomerases) that were shown in the upper area of reduced gels (Figs. 2, 3, 4, 5). As demonstrated in the immunoblot analysis (Fig. 1), we cannot exclude DTT-resistant states that may represent forms of proinsulin modified by non-disulfide bonds that still migrate to the upper area of the reduced gel. The addition of DTT here facilitated comparisons among these chases by providing a ^35^S-proinsulin pool, a significant fraction of which escaped clearance.

Antimycin, an inhibitor of ATP production and inducer of reactive oxygen species (ROS) in mitochondria [32], significantly suppressed proinsulin removal by uncharacterized mechanisms. Against the plateau in the DTT chase, about three-fourths of the proinsulin, mainly non-monomers and mostly naturally cleared, was retained with the addition of antimycin, whereas only about a third was retained in all other chases. The biggest ratio of non-monomers to monomers, noted as well in the antimycin chase, indicated that the addition of antimycin inhibited the conversion of proinsulin non-monomers to monomers in the maturation process (Fig. 4A, B; Table S10). Comparison of proinsulin monomer levels in the 12-minute chases with and without antimycin on non-reduced gel indicated maturation of over 20% of proinsulin monomers in this way (Fig. 4A). Concomitantly, a large fraction of proinsulin non-monomers that escaped from the antimycin block became monomers that could be processed energy independently or by utilizing the ATP present in cells.

Overall, a clearance route has been revealed in the early post-translational processing of proinsulin that is characterized by marked ATP/redox sensitivity (yin) counterbalanced by a maturation route that is partially dependent upon ATP (yang). The inhibition of proinsulin maturation, degradation, and movement herein exposed may be affected by energy poisons, such as antimycin, that influence proinsulin transport, a process observed in early indirect examinations [33], [34]. Moreover, a dual fate of proinsulin non-monomers for the yin-yang routes is revealed.

### Prolonged residual non-monomer fraction and rapid disposal (RD) of nascent proinsulin in the post-translational process

To ascertain how fast the abundant nascent proinsulin non-monomers disappear, we analyzed proinsulin kinetics in MIN6 β-cells chased at 3, 30, 60, and 60 minutes with antimycin after a 15-minute pulse. From the 3- to 60-minute chase, the proportion of proinsulin monomers in C-peptide immunoprecipitates increased from 54 to 83% (Fig. 5A, B; Table S14). However, this 83% can only be compared with the proportion of PC1/3 or pro-IAPP monomers at completion of 5- or 15-minute *de novo* synthesis (Figs. 3D, 4E; Tables S9, S11). Seventeen percent of nascent proinsulin is still in non-monomer states after a 75-minute course, during which regulated secretory proteins normally should already have been located in the immature secretory granules after a long trip through the ER, Golgi, and trans-Golgi network [15]. Proinsulin non-monomers showed a longer half-life than the control proteins in β-cells. Likewise, when pH in a test tube is neutral, proinsulin is prone to form amorphous precipitates and is less stable than insulin, and its unidentified intermediate folding states are suggested in *in vitro* studies outside the cell without the assistance of molecular chaperones [35], [36]. In a recent study, the property of matured insulin to unfold and oligomerize has been harnessed to achieve a glucose regulatory effort for treatment of diabetes in rodent models [37]. Thus, the long half-life could be linked to the primary amino acid sequence in addition to intracellular environments and may be physiologically required to facilitate processes and their regulation, such as the maturation, disposal, and (retro)transport in the quality control of the early secretory pathway [38]. As well, the gradually decreased proportion of non-monomers and gradually increased proportion of monomers in proinsulin composition during the 12- and 60-minute chases (Figs. 4,5) further showed the preferential processing of non-monomers during early post-translational processing.

Compared to the ^35^S-proinsulin plateau retained in the 60-minute chase with antimycin, up to 22% of ^35^S-proinsulin disappeared after the 3-minute chase; 52%, after the 30-minute chase; and 75%, after the 60-minute chase (Fig. 5A, C; Table S15). This result further supports the role of the disposal route in early proinsulin post-translational processing because numerous studies indicate that processing into insulin alone cannot fully explain such a significant drop in 60 minutes during the general 2-hour course of conversion [15]. As shown in the 12-minute chase (Fig. 4), 10 µM of antimycin cannot completely block proinsulin removal, and more proinsulin could actually be removed than our evidence shows. Even so, we validated removal of at least 20% of proinsulin by the mechanism of RD in these 2 chase courses (Figs. 4, 5) regardless of the different pulse times. Additionally, the inhibitive effect of antimycin shown in these 2 chase courses suggests a role of the mitochondria in proinsulin maturation and disposal, which provides new insights into the link between mitochondria and insulin secretion previously reviewed [39].

In eukaryotic cells, the lysosomal (autophagy) and proteasomal pathways are the 2 main routes by which cellular proteins are cleared. Up to 30% of nascent proteins are thought to be defective ribosomal products and can be rapidly degraded by proteasomes [40]. Under stress, a fraction of some secretory proteins can be removed by proteasomes in cytosol resulting from failure in passage of the ER translocon [41], [42] or by autophagy and ER-associated degradation after entry into the ER lumen [38], [43], [44]. It is unclear in proinsulin clearance whether RD is a completely novel process or an element of these revealed mechanisms, but there is no doubt that rapid clearance plays an active role in removing endogenous proinsulin in β-cells.

### Susceptibility of proinsulin maturation process to extra-/intracellular influences

The above study revealed the low relative folding rate/efficiency of proinsulin that underlies the susceptibility of proinsulin maturation to the influence of genetic disorder, DTT, antimycin, and other possible factors. To identify potential factors, we examined the effects of various reagents on proinsulin maturation. During a 60-minute post-translational process in which MIN6 β-cells were exposed to cycloheximide (Chx; 100 µg/mL, a protein synthesis inhibitor), we assessed the newly synthesized proinsulin states using C-peptide immunoblot. Results showed that compared with the untreated control, MIN6 β-cells treated with only Chx demonstrated significant disappearance of proinsulin (Fig. 6, Lane 2 versus Lane 1), indicating that the 60-minute inhibition of protein synthesis with Chx enabled processing (via disposal and maturation routes) of most proinsulin that was synthesized prior to the addition of the Chx. In contrast, proinsulin disappeared much more slowly when treatment with Chx was combined with tumor necrosis factor alpha (TNFα, 2 ng/mL), tunicamycin (1 ug/mL), thapsigargin (2 uM), verapamil (100 uM), nickel chloride (1 mM), iodoacetamide (1 mM), or hydrogen peroxide (H_2_O_2_, 250 uM) (Fig. 6, Lanes 3 to 9 versus Lane 2). The deceleration in protein maturation and disposal was marked by the accumulation of more non-monomers than monomers in most of these treatments. The predominant accumulation of non-monomers, shown earlier in the *Ins2^+/Akita^* islets and the antimycin treatment (Figs. 1, 2, 4, 5), was also noticeably induced by TNFα, thapsigargin, iodoacetamide, or H_2_O_2_ (Fig. 6). The less potent induction by tunicamycin most likely results from absence of the targeted process, N-linked glycosylation, in proinsulin maturation. The weak effect of nickel chloride demonstrated the seemingly insignificant role of the T-type calcium channel, the target of nickel chloride, in proinsulin maturation, although the channel may facilitate insulin secretion [45].

TNFα, a proinflammatory cytokine, has been implicated in the β-cell failure of T1D and T2D [46]. For instance, by reducing the glucose-stimulated influx of Ca^2+^, TNFα induced the impairment of glucose-stimulated insulin secretion (GSIS) [46]–[49] but did not affect insulin transcription [48]. Thapsigargin, an inhibitor of ER Ca^2+^ ATPase that induces UPR and ER stress by altering cytosolic and ER Ca^2+^ concentrations, can attenuate basal insulin release and GSIS [46], [50], [51]. Verapamil blocks the L-type calcium channel, the major subtype of calcium channels in β-cells [46], [52]; iodoacetamide can interfere with disulfide bond formation, a critical step in oxidative protein folding that generates ROS by-products [53], [54]; and H_2_O_2_, a highly reactive ROS that is enriched in normal β-cells (due partly to low levels of antioxidant enzymes), can impair cytosolic calcium handling [55]. Though the capacity of all these reagents to attenuate basal insulin release and/or 2-phase GSIS is known [13], [45]–[52], [54]–[56], we believe our study is the first that directly correlates these reagent-targeted molecules/pathways and the proinsulin maturation process. Such relationship would underlie the known negative effects on β-cells and especially serve as a basis for the vague mechanisms that underlie the affected second phase of GSIS. In addition, our preliminary studies have also indicated a role of glucolipotoxicity in T2D [2], [3], [8]–[10], [12]–[14] that affects PIHO (data not shown).

## Discussion

### Improved methodology enables determination of large fractional non-monomer states and low relative folding rate of proinsulin in normal β-cells

Proinsulin is the most abundant insulin precursor made in β-cells. The processing of proinsulin molecules involves a complex process of “folding.” Until recently, this was felt to occur very rapidly, resulting in formation of mature insulin molecules. In this study, we applied a number of protein extraction buffers, SPPs, antibodies, internal controls, reagents, and analytical principles (described in “Materials and Methods”) to improve C-peptide immunoblotting and pulse-chase approaches for analysis of cellular proinsulin states. Our use of the improved approaches allowed us to assess the fraction and folding nature of proinsulin monomers and non-monomers in islet/β-cell nascent or whole-cell protein pools. The results demonstrate that proinsulin preserves an aggregation-prone nature and a low relative folding rate that result in production of abundant non-natively folded non-monomer (i.e., aggregate) forms in normal mouse/human β-cells (Figs. 1, 2, 3, S1, S2, S3). The improved approaches will help further understanding of the folding and linked processes of cellular proinsulin and other proteins.

Of note, the non-natively folded proinsulin non-monomers showed lower affinity with the high conformation-dependent insulin antisera [30] than with C-peptide antisera (Figs. 2A, 2B, 3A, 3B). These results suggest that application of C-peptide antisera would be indeed helpful for adequate recovery/detection of proinsulin polypeptides including the non-natively folded forms in the study of proinsulin folding and related β-cell biology, although the insulin antisera are being used extensively in these aspects in current studies.

### Proinsulin maintains a homeostatic balance of non-natively and natively folded states in normal β-cells

Proinsulin is proven to be inherent with an aggregation-prone nature and a low relative folding rate but it is the most abundant insulin precursor made in the pancreatic β-cells [22]–[24]. So how does the β-cell normally control proinsulin early post-translational processing? The results of our pulse-chase studies indicate that the preferential removal of dual fate non-natively folded non-monomers of proinsulin serves as an adaptive mechanism for flow control of secretory proteins. Proinsulin thus maintains a homeostatic balance of natively and non-natively folded states (i.e., proinsulin homeostasis, PIHO) in normal β-cells as a result of the integration of disposal and maturation processes (Figs. 4,5).

Interestingly, we found the greater abundance of unidentified molecular helpers in the non-monomer states of nascent proinsulin than in pro-IAPP and PC1/3 non-monomer states (as a larger ratio of upper-area signal to proinsulin monomer signal in insulin and C-peptide immunoprecipitates on reduced gels) (Figs. 3, 4). These observations show the recovery of varied levels of molecular helpers of diverse composition in the immunoprecipitates of different nascent secretory proteins. These observations also support a recent notion that the folding of diverse secretory proteins is assisted by “public” and “private” chaperones that are enriched in specialized subregions to ensure the efficiency and fidelity of protein folding [38]. In proinsulin non-monomer states, such chaperones seem to be remarkably enriched despite the fact that chaperones of the folding and/or degradation machineries are mostly shared by diverse substrates. In addition, variations in the levels and/or composition of molecular helpers between normal and *Ins2^+/Akita^* islets (Fig. 2C) suggest that those differences could serve as a rheostat to influence proinsulin maturation, disposal, and flow in the early secretory pathway of (ab)normal β-cells. Collectively, these observations demonstrate that the control of precursor maturation and disposal is an early regulative mechanism in the normal insulin production of β-cells. In this context, molecular helpers located in the non-monomer states of proinsulin may facilitate regulations of the maturation and disposal of insulin precursor.

### The link between β-cell (dys)function, diabetes, and PIHO

The relative slow completed folding process of proinsulin, which is exposed in this study, provides not only a site for early regulations in the normal insulin biosynthesis but also a basis for the susceptibility of PIHO to various stresses. As shown in our immunoblotting and pulse- chase studies (Figs. 1, 4, 5, 6), PIHO disorders were induced by the *Ins2^+/Akita^* genetic defect, cellular energy and calcium changes, ER and reductive or oxidative stress, and insults by thiol reagent and cytokine. Most of these factors are associated with diabetes [1]–[15], [39], [45], [46]. Moreover, we have recently found that PIHO disorders occur also in non-obese diabetic (NOD) and db/db mice (unpublished data) that are typical models representing for the main forms of diabetes in humans. Our recent studies have also revealed that the primarily disturbed PIHO results in a number of toxic consequences with known association to β-cell failure in general forms of diabetes. These include the atypical proinsulin disposal and processing, ER and oxidative stress, mitochondrial abnormalities, and β-cell depletion [16], [17] (unpublished observations).

**Figure 7:**
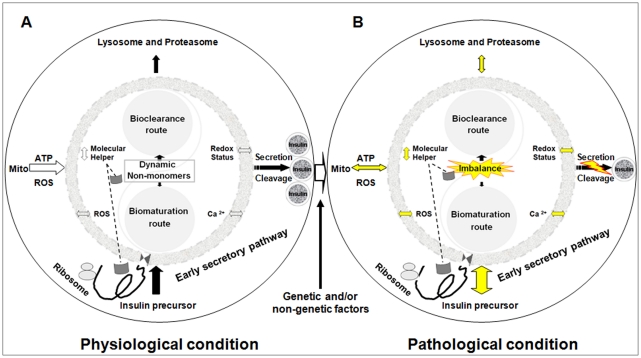
Perturbation of PIHO contributes to β-cell failure and diabetes. (A) Proinsulin maintains a homeostatic balance of natively and non-natively folded states (i.e., proinsulin homeostasis, PIHO) in normal β-cells as a result of the integration of maturation and degradation processes. Normal β-cells can control insulin production by early regulations of its precursor maturation and disposal in response to extra- and intracellular influences. Molecular helpers located in non-monomer states facilitate proinsulin maturation and/or disposal. (B) Based on our recent studies, we have suggested that the perturbation of PIHO is an early defect of diabetes induced by varied factors. Over time, vicious cycles of the PIHO disorders and linked pathways and/or compartments would be established that could contribute to the development of β-cell damage and diabetes. Yellow indicates possible abnormalities. Left-to-right or up-and-down arrows show possible 2-way alterations/influences. Mito, mitochondria.

All these findings help us understand why proinsulin is an important molecule linked to β-cell dysfunction in addition to being the natural precursor to insulin. Based on our accumulated evidence, we hypothesized a model that PIHO, an essential post-translational regulation mechanism in insulin biosynthesis, may critically link to diabetes (Fig. 7). We propose that the early control of precursor maturation and disposal is an essential mechanism in controlling insulin production and secretion in normal β-cells (Fig. 7A). Consequently, the imbalance of proinsulin maturation and disposal (induced by various factors) results in a number of consequences that contribute to the development of β-cell failure and diabetes (Fig. 7B).

In this study, we establish an approach for assessing proinsulin maturation and disposal in cells that disclosed some fundamental features of proinsulin folding and the direct correlation among various diabetes-related risk factors. However, the molecular mechanisms remain unclear. Further investigations are underway. This study also provides the first experimental evidence for hypothesizing polypeptide homeostasis in normal cells (PHIC). Under certain conditions, molecular interactions in biological processes reveal PHIC with detectable (non-)natively folded protein states, such as those states in this study. Perturbations of PHIC result in various diseases associated with atypical protein folding, disposal, and toxic consequences [6], [10], [27], [31]. The use of PHIC to determine (im)balances of natively and non-natively folded states of disease-associated proteins, such as molecules with low folding rate or fibrillation-prone nature, will help identify true biological defects under pathological conditions that could result in improved interventions and treatments.

## Materials and Methods

For further details, please see our Text S1 Supplemental Experimental Procedures, which are published as Supplemental Data on the *PLoS ONE* web site.

### Ethics statement

All animal and tissue sample experiments were performed in accordance with the guidelines of the National Institutes of Health and The Ohio State University with procedures (2007A0040 and 2010A0024) approved by the Institutional Animal Care and Use Committee (IACUC) of the university.

### Materials

We obtained antibodies, chemicals, mice, and human islets from companies and other investigators, which are detailed in the Text S1 Supplemental Experimental Procedures.

### Islet preparation, cell culture, and pulse-chase

Unless specifically stated, we conducted all operations and maintained all materials away from reducing reagents. Islet isolation and islet and cell line culture were described previously [16], [18]. Briefly, for treatment, cells were incubated in 35-mm dishes in DMEM (25.5 mM glucose) medium supplemented with 10% fetal bovine serum plus (FBS) at 37°C with 5% CO_2_/95% O_2_ until 80 to 90% confluence. Isolated islets were cultured overnight in 10% FBS/RPMI 1640 (11 mM glucose) until treatment.

After 30 minutes' preincubation in Met-free DMEM for MIN6 β-cells or Met and/or cysteine-free RPMI 1640 medium for islets, mouse islets or MIN6 cells were labeled with ^35^S-Met, and human islets were labeled with ^35^S-Met/^35^S-Cys in the same media. We conducted the various chase tests using pre-balanced complete DMEM/10% FBS media with or without agents. After pulse and/or chase incubations, islets and MIN6 β-cells were quickly washed twice with PBS containing 20 mM N-ethylmaleimide and immediately lysed in the IP buffer and/or frozen at −80°C.

### Protein extraction buffers and sample preparations for immunoblotting and immunoprecipitation

To extract protein, we utilized RIPA, tricine gel sample (Bio-Rad), and IP buffers [16], [29]. The SPP procedures, IP tests, and protein samples for SDS-PAGE are detailed in the Text S1 Supplemental Experimental Procedures.

### SDS-PAGE

We boiled protein samples for 10 minutes in tricine gel sample buffer with and without 100 mM of DTT for SDS-PAGE. Tricine-SDS-PAGE gels without urea were applied for all other tests in this article except the one with urea (Fig. 3A) and the 7.5% Laemmli SDS-PAGE (Fig. 4D). Gel concentrations (%T, % C; T denotes the total percentage of concentration of both acrylamide and bisacrylamide; C denotes the percentage of concentration of bisacrylamide relative to total concentration) are described in figure legends.

### Immunoblotting and radioautography

We performed immunoblotting following standard procedures using antibodies against C-peptide or tubulin. For autoradiography, we dried membrane and fixed gels with labeled materials for exposure on x-films.

### Quantitative analysis of immunoreactivity and radioactivity

To quantify the density and radioactivity of protein bands (area and/or gel slices), we used National Institutes of Health (NIH) Image J software and/or liquid scintillation counter and gamma counter (Beckman Coulter, Inc., Brea, CA, USA).

### Methods of calculating the proportions of proinsulin or control protein monomers and non-monomers

In general, the amount of protein monomer(s) in a reduced SDS-PAGE/membrane (by adding DTT) is accepted as the total level of protein in loaded samples. Resolution of equal amounts of protein in individual samples by SDS-PAGE under non-reduced and reduced conditions permits calculation of the fractions of protein monomers and non-monomers (referred to as DTT-sensitive forms) in individual samples. The resolution is possible because conversion of protein from its DTT-sensitive non-monomer states to monomers by the effect of DTT yields different protein monomer levels between the two conditions. We calculated the proportions of proinsulin or control protein monomers and non-monomers shown in the following sections by the formulas: monomers (%) = (monomer level under non-reduced condition)×100%/(monomer level under reduced condition); non-monomers (%) = (monomer level under reduced condition−monomer level under non-reduced condition)×100%/(monomer level under reduced condition); or non-monomers (%) = 100%−monomers (%).

### Data analysis

Data are presented as the mean ± standard deviation (SD; n = 3 to 6). We assessed statistical significance (*P<0.05; **P<0.005) by Student's t-test (2-tailed) or analysis of variance if appropriate.

### Supplemental data

Supplemental data, including Text S1 supplemental experimental procedures, are presented with 3 figures and 15 tables on the *PLoS ONE* web site.

## Supporting Information

Figure S1 **Molecular weights of the clearly resolved states of insulin precursor shown in** **figure 1** **(A) and the states of insulin precursor in cloned mouse β-cells (B).** (A) Molecular weights of the well resolved proinsulin states were calculated by comparing their electrophoretic mobility with those of the SeeBlue® Plus2 protein markers (Invitrogen). The molecular weights of the smears can be inferred by the shown molecular weights of protein markers. (B) Whole-cell proteins of *Ins2^+/+^*, *Ins2^+/Akita^*, and MIN6 β-cells were extracted directly in the tricine sample buffer by SPP-B, resolved by tricine-SDS-PAGE without urea (20%T, 5% C), and then subjected to C-peptide immunoblotting analysis.
(TIF)

Figure S2 **Completely folded ^125^I-proinsulin in islet protein extracts through immunoprecipitation and electrophoresis does not form significant aggregates.** We added ^125^I-proinsulin monomer marker (Linco) to the immunoprecipitation (IP) buffer and subjected *Ins2^+/+^* and *Ins2^+/Akita^* islet proteins extracted in this IP buffer to IP with insulin and C-peptide antisera. Equal amounts of individual immunoprecipitates were resolved by 10% non-reduced and reduced tricine-SDS-PAGE. Gel radioautograph showed that no significant aggregation of the ^125^I-proinsulin itself and/or with islet proteins (e.g., endogenous proinsulin) occurred through the IP and electrophoresis.
(TIF)

Figure S3 **The islet nascent proteins with a small fraction of non-monomers after 30-min** ***de novo*** **synthesis.** Mouse islets were labeled with ^35^S–Met for 30 minutes, and cellular proteins were then subjected to IP with antisera against to the proteins listed above. Equal amounts of individual immunoprecipitates were resolved by non-reduced and reduced SDS-PAGE for autoradiography. The monomer and non-monomer proportions of proteins in immunoprecipitates were calculated using the method introduced in “Materials and Methods.” The calculated results indicated that the non-monomer proportions of the above shown proteins were all less than 20%.
(TIF)

Table S1 **Proportions of individual states of insulin precursor in** **Figure 1A** **.**
(PDF)

Table S2 **Percentage of proinsulin monomers under non-reduced versus reduced condition in** **Figure 1A** **.**
(PDF)

Table S3 **Proportions of proinsulin monomers and non-monomers in the** ***Ins2^+/+^*** **and** ***Ins2^+/Akita^*** **islets in** **Figure 1A** **.**
(PDF)

Table S4 **Proportions of nascent proinsulin monomers and non-monomers precipitated by insulin (Ins) or C-peptide (Cp) antisera from mouse islets labeled for 30 minutes.**
(PDF)

Table S5 **Proportions of nascent proinsulin monomers and non-monomers precipitated by C-peptide antisera from** ***Ins2^+/+^*** **and** ***Ins2^+/Akita^*** **islets labeled for 45 minutes.**
(PDF)

Table S6 **Proportions of nascent proinsulin monomers and non-monomers in human islets labeled for 30 minutes.**
(PDF)

Table S7 **Completely folded ^125^I-proinsulin in** ***Ins2^+/+^*** **and** ***Ins2^+/Akita^*** **islet protein extracts that was subjected to IP and electrophoresis did not form significant aggregates.**
(PDF)

Table S8 **Proportions of proinsulin monomers and nom-monomers in C-peptide (Cp) or insulin (Ins) immunoprecipitates obtained from MIN6 β-cells labeled for 5, 15, or 30 minutes.**
(PDF)

Table S9 **Proportions of islet amyloid polypeptide precursor monomers and nom-monomers in MIN6 β-cells labeled for 5, 15, or 30 minutes.**
(PDF)

Table S10 **Proportions of nascent proinsulin monomers and nom-monomers in MIN6 β-cells chased for the indicated times with/without antimycin, DTT, or GSSG after a 5-min pulse.**
(PDF)

Table S11 **Proportions of nascent PC1/3 monomers and nom-monomers in MIN6 β-cells chased for the indicated times with/without antimycin, DTT, or GSSG after a 5-min pulse.**
(PDF)

Table S12 **Relative levels of nascent proinsulin in MIN6 β-cells chased for the indicated times with/without antimycin, DTT, or GSSG after a 5-min pulse.**
(PDF)

Table S13 **Relative levels of nascent PC1/3 in MIN6 β-cells chased for the indicated times with/without antimycin, DTT, or GSSG after a 5-min pulse.**
(PDF)

Table S14 **Proportions of nascent proinsulin monomers and nom-monomers in MIN6 β-cells chased for the indicated times with/without antimycin after a 15-min pulse.**
(PDF)

Table S15 **Relative levels of nascent proinsulin in MIN6 β-cells chased for the indicated times with/without antimycin after a 15-min pulse.**
(PDF)

Text S1 **Supplemental Experimental Procedures.**
(DOC)
