# Supplementary figures and images for: Control of Precursor Maturation and Disposal Is an Early Regulative Mechanism in the Normal Insulin Production of Pancreatic β-Cells
Source: PLoS One. 2011 Apr 29;6(4):e19446. doi: 10.1371/journal.pone.0019446 (PMC3084858; doi:10.1371/journal.pone.0019446)

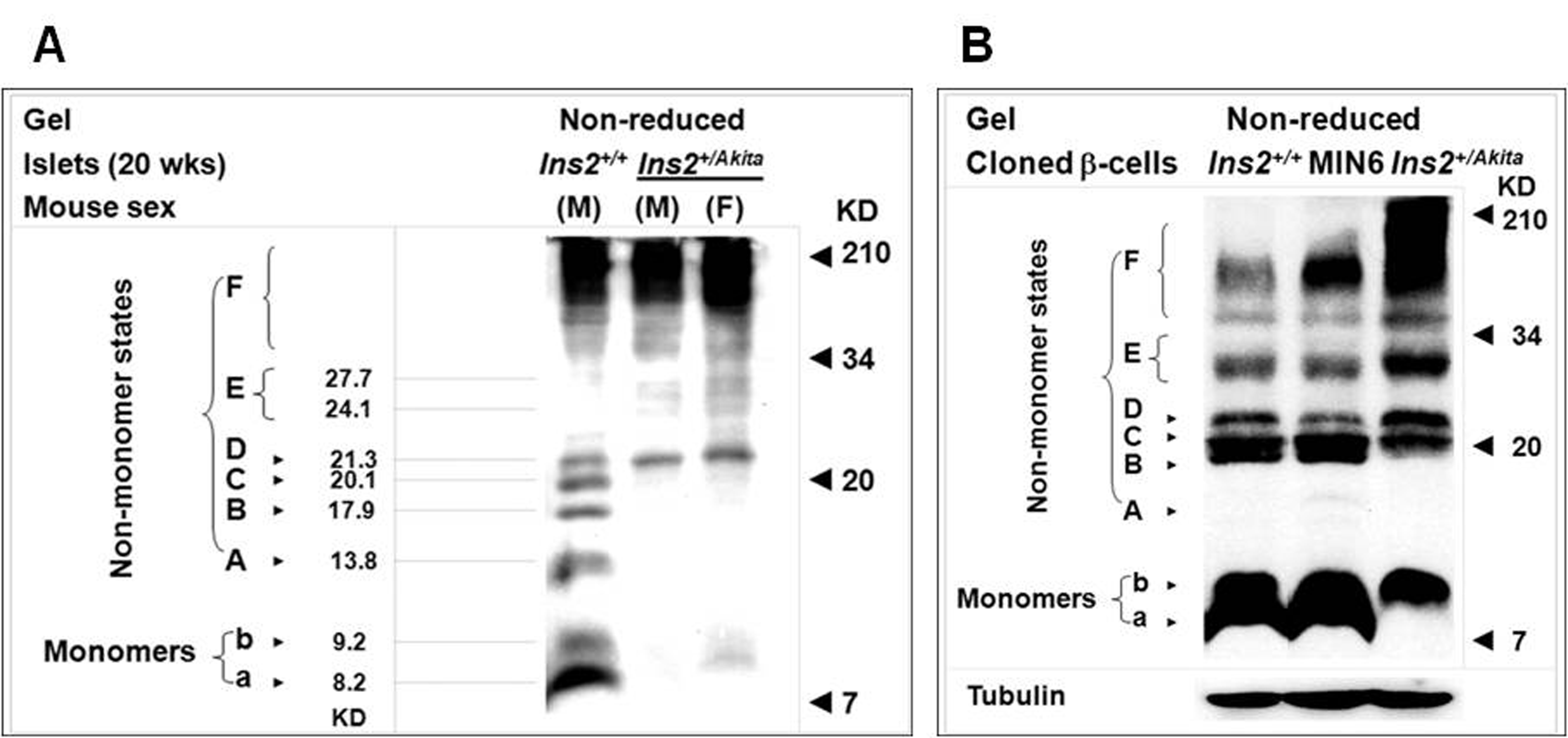

Supplement: Figure S1 — Molecular weights of the clearly resolved states of insulin precursor shown in figure 1 (A) and the states of insulin precursor in cloned mouse β-cells (B). (A) Molecular weights of the well resolved proinsulin states were calculated by comparing their electrophoretic mobility with those of the SeeBlue® Plus2 protein markers (Invitrogen). The molecular weights of the smears can be inferred by the shown molecular weights of protein markers. (B) Whole-cell proteins of Ins2+/+, Ins2+/Akita, and MIN6 β-cells were extracted directly in the tricine sample buffer by SPP-B, resolved by tricine-SDS-PAGE without urea (20%T, 5% C), and then subjected to C-peptide immunoblotting analysis. (TIF) [file pone.0019446.s001.tif]

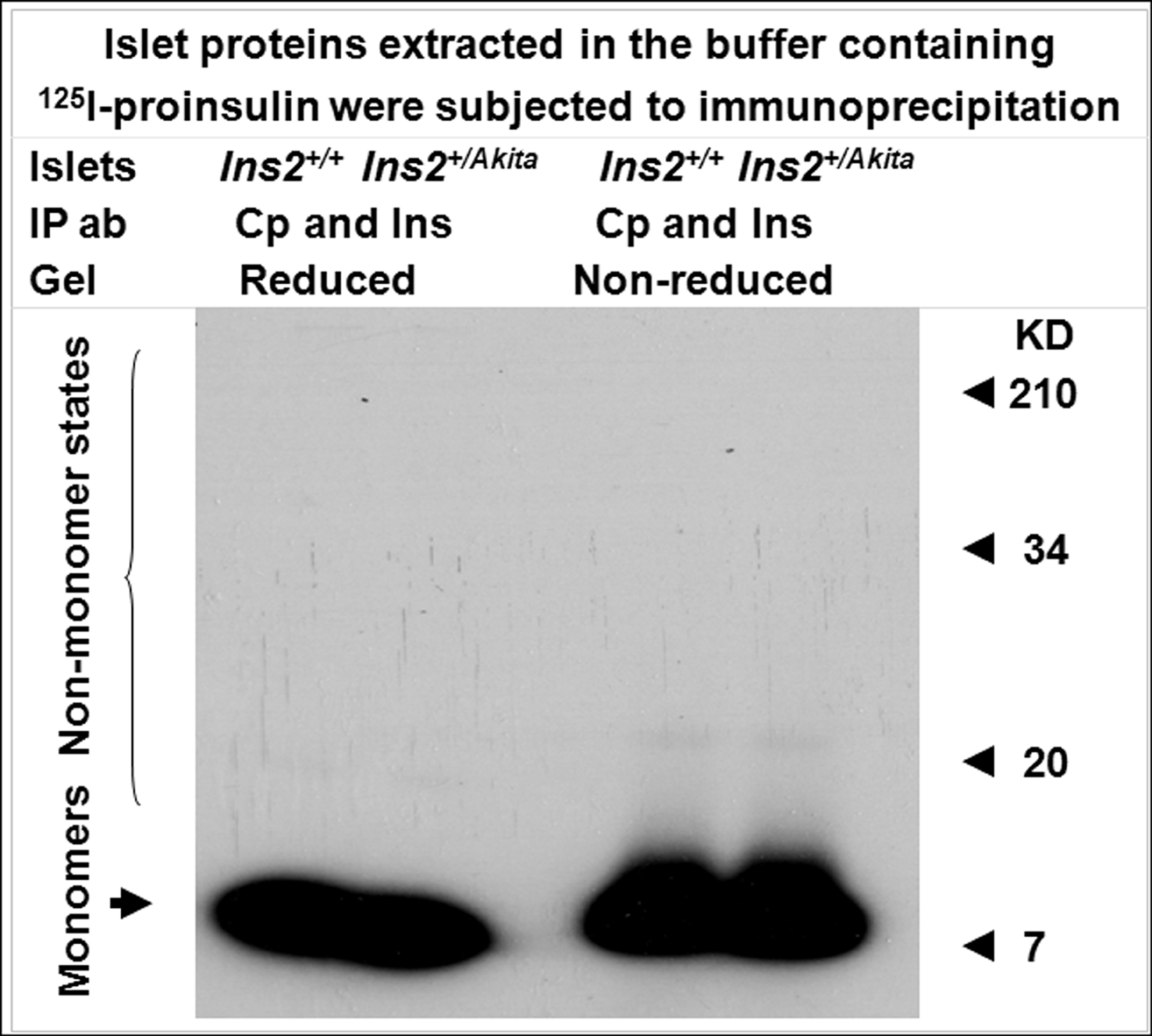

Supplement: Figure S2 — Completely folded 125I-proinsulin in islet protein extracts through immunoprecipitation and electrophoresis does not form significant aggregates. We added 125I-proinsulin monomer marker (Linco) to the immunoprecipitation (IP) buffer and subjected Ins2+/+ and Ins2+/Akita islet proteins extracted in this IP buffer to IP with insulin and C-peptide antisera. Equal amounts of individual immunoprecipitates were resolved by 10% non-reduced and reduced tricine-SDS-PAGE. Gel radioautograph showed that no significant aggregation of the 125I-proinsulin itself and/or with islet proteins (e.g., endogenous proinsulin) occurred through the IP and electrophoresis. (TIF) [file pone.0019446.s002.tif]

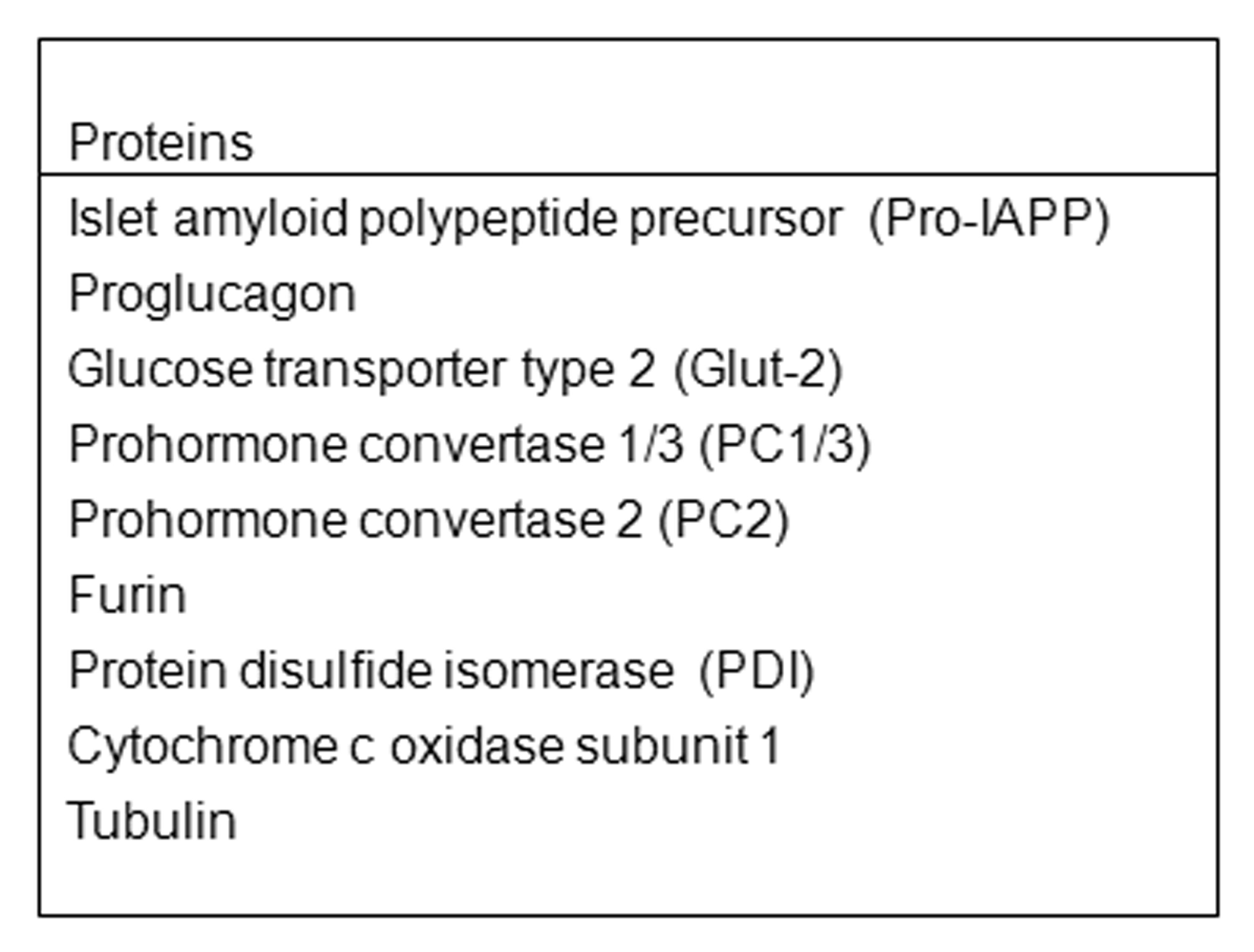

Supplement: Figure S3 — The islet nascent proteins with a small fraction of non-monomers after 30-min de novo synthesis. Mouse islets were labeled with 35S–Met for 30 minutes, and cellular proteins were then subjected to IP with antisera against to the proteins listed above. Equal amounts of individual immunoprecipitates were resolved by non-reduced and reduced SDS-PAGE for autoradiography. The monomer and non-monomer proportions of proteins in immunoprecipitates were calculated using the method introduced in “Materials and Methods.” The calculated results indicated that the non-monomer proportions of the above shown proteins were all less than 20%. (TIF) [file pone.0019446.s003.tif]
