# Supplementary material for: Control of Precursor Maturation and Disposal Is an Early Regulative Mechanism in the Normal Insulin Production of Pancreatic β-Cells
Source: PLoS One. 2011 Apr 29;6(4):e19446. doi: 10.1371/journal.pone.0019446 (PMC3084858; doi:10.1371/journal.pone.0019446)
Supplement: Table S1 — Proportions of individual states of insulin precursor in Figure 1A . (PDF) [file pone.0019446.s004.pdf]

Table S1. Proportions of individual states of insulin precursor in Figure 1A

Percentage	Mean	Mean	Mean	SD	SD	SD	P	P
Islets (20 wks of age)	Control (M)	Akita (M)	Akita (F)	Control (M)	Akita (M)	Akita (F)	C vs. A (M) (M)	C vs. A (M) (F)
Non-reduced Condition								
Monomers (a and b)	29.5	2.9	3.0	6.1	4.4	1.6	<0.05	<0.05
Monomer a	17.4	0.5	0.4	1.5	0.3	0.3	<0.05	<0.05
Monomer b	12.1	2.5	2.6	4.8	4.1	1.4	0.005	<0.05
Non-monomers (A-F)	70.5	97.1	97	6.1	4.4	1.6	<0.05	<0.05
A	6.1	0.0	0.0	3.4	0.0	0.0	<0.05	<0.05
B	3.3	0.1	0.3	1.1	0.2	0.4	<0.05	<0.05
C	5.0	0.7	1.3	0.5	0.7	0.4	<0.05	<0.05
D	3.0	6.9	7.3	1.3	1.7	1.0	<0.05	<0.05
E	4.4	10.3	10.7	2.1	5.7	0.8	0.04	<0.05
F	48.7	79.0	77.4	1.4	10.5	2.6	<0.05	<0.05
Reduced Condition								
Monomers (a and b)	90.2	95.7	95.8	2.0	16.5	16.2	0.7	0.8
Monomer a	41.7	5	3.4	9.6	1.5	2.1	<0.05	<0.05
Monomer b	48.5	90.7	92.4	11.6	16.1	43.3	<0.05	<0.05
Non-monomers (A-F)	9.8	4.3	4.2	5.6	4.2	3.1	0.1	0.2

Control (C): *Ins2*$^{+/+}$; Akita (A): *Ins2*$^{+/Akita}$; M, male; F, female. Data are shown in Figure 1B.
