# Supplementary material for: Control of Precursor Maturation and Disposal Is an Early Regulative Mechanism in the Normal Insulin Production of Pancreatic β-Cells
Source: PLoS One. 2011 Apr 29;6(4):e19446. doi: 10.1371/journal.pone.0019446 (PMC3084858; doi:10.1371/journal.pone.0019446)
Supplement: Table S2 — Percentage of proinsulin monomers under non-reduced versus reduced condition in Figure 1A . (PDF) [file pone.0019446.s005.pdf]

Table S2. Percentage of proinsulin monomers under non-reduced versus reduced condition in Figure 1A

Percentage	Gel Condition	Control	Akita (M)	Akita (F)
Mean	Non-Reduced	71.1	4.7	9.8
Mean	Reduced	100	100	100
SD	Non-Reduced	3.6	3.3	6.8
SD	Reduced	13.5	11.0	17.0
P (Non-Reduced vs. Reduced)		0.02	<0.005	<0.005

Control: *Ins2*$^{+/+}$; Akita: *Ins2*$^{+/Akita}$; M, male; F, female. Data are shown in Figure 1C.
