# Supplementary material for: Control of Precursor Maturation and Disposal Is an Early Regulative Mechanism in the Normal Insulin Production of Pancreatic β-Cells
Source: PLoS One. 2011 Apr 29;6(4):e19446. doi: 10.1371/journal.pone.0019446 (PMC3084858; doi:10.1371/journal.pone.0019446)
Supplement: Table S3 — Proportions of proinsulin monomers and non-monomers in the Ins2+/+ and Ins2+/Akita islets in Figure 1A . (PDF) [file pone.0019446.s006.pdf]

Table S3. Proportions of proinsulin monomers and non-monomers in the *Ins2*$^{+/+}$ and *Ins2*$^{+/Akita}$ islets in Figure 1A

Percentage	State	Control (M)	Akita (M)	Akita (F)
Mean	Monomers	71.1	4.7	9.8
Mean	Non-monomers	28.9	95.3	90.2
SD	Monomers	3.6	3.3	6.8
SD	Non-monomers	3.6	3.3	6.8
P (Control vs. Akita)			<0.005	<0.005

Control: *Ins2*$^{+/+}$; Akita: *Ins2*$^{+/Akita}$; M, male; F, female. Data are shown in Figure 1D.
