# Supplementary material for: Control of Precursor Maturation and Disposal Is an Early Regulative Mechanism in the Normal Insulin Production of Pancreatic β-Cells
Source: PLoS One. 2011 Apr 29;6(4):e19446. doi: 10.1371/journal.pone.0019446 (PMC3084858; doi:10.1371/journal.pone.0019446)
Supplement: Table S4 — Proportions of nascent proinsulin monomers and non-monomers precipitated by insulin (Ins) or C-peptide (Cp) antisera from mouse islets labeled for 30 minutes. (PDF) [file pone.0019446.s007.pdf]

Table S4. Proportions of nascent proinsulin monomers and non-monomers precipitated by insulin (Ins) or C-peptide (Cp) antisera from mouse islets labeled for 30 minutes

Percentage	Proinsulin State	Ins IP	Cp IP
Mean	Monomers	58.2	39.7
Mean	Non-monomers	41.8	60.3
SD	Monomers	4.2	7.6
SD	Non-monomers	4.2	7.6
P (Monomers; Ins IP vs. Cp IP)			<0.005
P (Non-monomers; Ins IP vs. Cp IP)			<0.005

(Shown in Figure 2B)
