# Supplementary material for: Control of Precursor Maturation and Disposal Is an Early Regulative Mechanism in the Normal Insulin Production of Pancreatic β-Cells
Source: PLoS One. 2011 Apr 29;6(4):e19446. doi: 10.1371/journal.pone.0019446 (PMC3084858; doi:10.1371/journal.pone.0019446)
Supplement: Table S5 — Proportions of nascent proinsulin monomers and non-monomers precipitated by C-peptide antisera from Ins2+/+ and Ins2+/Akita islets labeled for 45 minutes. (PDF) [file pone.0019446.s008.pdf]

Table S5. Proportions of nascent proinsulin monomers and non-monomers precipitated by C-peptide antisera from *Ins2*$^{+/+}$ and *Ins2*$^{+/Akita}$ islets labeled for 45 minutes

Percentage	Proinsulin State	Control	Akita (M)
Mean	Monomers	71.0	22.8
Mean	Non-monomers	29.0	77.2
SD	Monomers	14.5	5.0
SD	Non-monomers	14.5	5.0
P (Monomers of Control vs. Akita)			<0.005
P (Non-monomers of Control vs. Akita)			<0.005

(Shown in Figure 2D)
