# Supplementary material for: Control of Precursor Maturation and Disposal Is an Early Regulative Mechanism in the Normal Insulin Production of Pancreatic β-Cells
Source: PLoS One. 2011 Apr 29;6(4):e19446. doi: 10.1371/journal.pone.0019446 (PMC3084858; doi:10.1371/journal.pone.0019446)
Supplement: Table S6 — Proportions of nascent proinsulin monomers and non-monomers in human islets labeled for 30 minutes. (PDF) [file pone.0019446.s009.pdf]

Table S6. Proportions of nascent proinsulin monomers and non-monomers in human islets labeled for 30 minutes

Percentage	Proinsulin State	Ins/Cp IP
Mean	Monomers	65.7
Mean	Non-monomers	34.3
SD	Monomers	3.4
SD	Non-monomers	3.4

(Shown in Figure 2F)
