# Supplementary material for: Control of Precursor Maturation and Disposal Is an Early Regulative Mechanism in the Normal Insulin Production of Pancreatic β-Cells
Source: PLoS One. 2011 Apr 29;6(4):e19446. doi: 10.1371/journal.pone.0019446 (PMC3084858; doi:10.1371/journal.pone.0019446)
Supplement: Table S7 — Completely folded 125I-proinsulin in Ins2+/+ and Ins2+/Akita islet protein extracts that was subjected to IP and electrophoresis did not form significant aggregates. (PDF) [file pone.0019446.s010.pdf]

Table S7. Completely folded ^{125}I-proinsulin in *Ins2*$^{+/+}$ and *Ins2*$^{+/Akita}$ islet protein extracts that was subjected to IP and electrophoresis did not form significant aggregates

Percentage	Gel Condition	Proinsulin State	Control	Akita
Mean	Non-reduced	Monomers	99.5	98.9
Mean	Non-reduced	Non-monomers	0.5	0.1
Mean	Reduced	Monomers	99.8	99.8
Mean	Reduced	Non-monomers	0.2	0.2
SD	Non-reduced	Monomers	2.0	2.3
SD	Non-reduced	Non-monomers	2.0	2.3
SD	Reduced	Monomers	5.3	7.4
SD	Reduced	Non-monomers	5.3	7.4

(Shown in Figure S2)
