# Supplementary material for: Control of Precursor Maturation and Disposal Is an Early Regulative Mechanism in the Normal Insulin Production of Pancreatic β-Cells
Source: PLoS One. 2011 Apr 29;6(4):e19446. doi: 10.1371/journal.pone.0019446 (PMC3084858; doi:10.1371/journal.pone.0019446)
Supplement: Table S8 — Proportions of proinsulin monomers and nom-monomers in C-peptide (Cp) or insulin (Ins) immunoprecipitates obtained from MIN6 β-cells labeled for 5, 15, or 30 minutes. (PDF) [file pone.0019446.s011.pdf]

Table S8. Proportions of proinsulin monomers and nom-monomers in C-peptide (Cp) or insulin (Ins) immunoprecipitates obtained from MIN6 β-cells labeled for 5, 15, or 30 minutes

MIN6	Proinsulin State	Cp-P5	Cp-P15	Cp-P30	Ins-P5	Ins-P15	Ins-P30
Mean	Monomers	41.3	42.5	49.1	63.5	73.4	76.1
Mean	Non-monomers	58.7	57.5	50.9	36.5	26.6	23.9
SD	Monomers	9.2	9.0	14.0	18.8	17.3	15.0
SD	Non-monomers	9.2	9.0	14.0	18.8	17.3	15.0
P (p5 vs. others)			0.83	0.28		0.362	0.23

P5, 5-min pulse; P15, 15-min pulse; P30; 30-min pulse. Data are shown in Figure 3B.
