# Supplementary material for: Control of Precursor Maturation and Disposal Is an Early Regulative Mechanism in the Normal Insulin Production of Pancreatic β-Cells
Source: PLoS One. 2011 Apr 29;6(4):e19446. doi: 10.1371/journal.pone.0019446 (PMC3084858; doi:10.1371/journal.pone.0019446)
Supplement: Table S9 — Proportions of islet amyloid polypeptide precursor monomers and nom-monomers in MIN6 β-cells labeled for 5, 15, or 30 minutes. (PDF) [file pone.0019446.s012.pdf]

Table S9. Proportions of islet amyloid polypeptide precursor monomers and nom-monomers in MIN6 β-cells labeled for 5, 15, or 30 minutes

Percentage	ProIAPP State	P5	P15	P30
Mean	Monomers	76.5	83.6	88.3
Mean	Non-monomers	23.5	16.4	11.7
SD	Monomers	4.0	2.1	3.5
SD	Non-monomers	4.0	2.1	3.5
P (P5 vs. others)			0.006	<0.005

P5, 5-min pulse; P15, 15-min pulse; P30; 30-min pulse. Data are shown in Figure 3D.
