# Supplementary material for: Control of Precursor Maturation and Disposal Is an Early Regulative Mechanism in the Normal Insulin Production of Pancreatic β-Cells
Source: PLoS One. 2011 Apr 29;6(4):e19446. doi: 10.1371/journal.pone.0019446 (PMC3084858; doi:10.1371/journal.pone.0019446)
Supplement: Table S10 — Proportions of nascent proinsulin monomers and nom-monomers in MIN6 β-cells chased for the indicated times with/without antimycin, DTT, or GSSG after a 5-min pulse. (PDF) [file pone.0019446.s013.pdf]

Table S10. Proportions of nascent proinsulin monomers and nom-monomers in MIN6 β-cells chased for the indicated times (minutes) with/without antimycin, DTT, or GSSG after a 5-min pulse

Percentage	Proinsulin State	C3	C6	C12	C12-Antimycin	C12-DTT	C12-GSSG
Mean	Monomers	46.3	49.9	53.4	16.5	80.0	53.7
Mean	Non-monomers	53.7	50.1	46.6	83.5	20.0	46.3
SD	Monomers	5.2	4.8	5.0	1.9	7.2	7.4
SD	Non-monomers	5.2	4.8	5.0	1.9	7.2	7.4
P (c3 vs. others)			0.3	0.04	<0.005	<0.005	0.08
P(c12 vs. others)		0.04	0.2		<0.005	<0.005	0.9

(Shown in Figure 4B)
