# Supplementary material for: Control of Precursor Maturation and Disposal Is an Early Regulative Mechanism in the Normal Insulin Production of Pancreatic β-Cells
Source: PLoS One. 2011 Apr 29;6(4):e19446. doi: 10.1371/journal.pone.0019446 (PMC3084858; doi:10.1371/journal.pone.0019446)
Supplement: Table S11 — Proportions of nascent PC1/3 monomers and nom-monomers in MIN6 β-cells chased for the indicated times with/without antimycin, DTT, or GSSG after a 5-min pulse. (PDF) [file pone.0019446.s014.pdf]

Table S11. Proportions of nascent PC1/3 monomers and nom-monomers in MIN6 β-cells chased for the indicated times (minutes) with/without antimycin, DTT, or GSSG after a 5-min pulse

Percentage	PC1/3 State	C3	C6	C12	C12-Antimycin	C12-DTT	C12-GSSG
Mean	Monomers	85.0	89.4	90.0	94.7	95.6	94.4
Mean	Non-monomers	15.0	10.6	10.0	5.3	4.4	5.6
SD	Monomers	2.8	2.7	1.8	1.0	3.0	2.6
SD	Non-monomers	2.8	2.7	1.8	1.0	3.0	2.6
P (12C vs. Others)		<0.005	0.5		<0.005	<0.005	0.2

(Shown in Figure 4E)
