# Supplementary material for: Control of Precursor Maturation and Disposal Is an Early Regulative Mechanism in the Normal Insulin Production of Pancreatic β-Cells
Source: PLoS One. 2011 Apr 29;6(4):e19446. doi: 10.1371/journal.pone.0019446 (PMC3084858; doi:10.1371/journal.pone.0019446)
Supplement: Table S12 — Relative levels of nascent proinsulin in MIN6 β-cells chased for the indicated times with/without antimycin, DTT, or GSSG after a 5-min pulse. (PDF) [file pone.0019446.s015.pdf]

Table S12. Relative levels of nascent proinsulin in MIN6 β-cells chased for the indicated times (minutes) with/without antimycin, DTT, or GSSG after a 5-min pulse

Percentage	C3	C6	C12	C12-Antimycin	C12-DTT	C12-GSSG
Mean	35.1	36.6	28.7	74.2	100.0	27.3
SD	3.0	3.4	2.6	6.7	10.3	2.9
P (c12 DTT vs. others)	<0.005	<0.005	<0.005	<0.005		<0.005
P (C12 vs. others)	<0.005	<0.005		<0.005	<0.005	0.4

(Shown in Figure 4C)
