# Supplementary material for: Control of Precursor Maturation and Disposal Is an Early Regulative Mechanism in the Normal Insulin Production of Pancreatic β-Cells
Source: PLoS One. 2011 Apr 29;6(4):e19446. doi: 10.1371/journal.pone.0019446 (PMC3084858; doi:10.1371/journal.pone.0019446)
Supplement: Table S13 — Relative levels of nascent PC1/3 in MIN6 β-cells chased for the indicated times with/without antimycin, DTT, or GSSG after a 5-min pulse. (PDF) [file pone.0019446.s016.pdf]

Table S13. Relative levels of nascent PC1/3 in MIN6 β-cells chased for the indicated times (minutes) with/without antimycin, DTT, or GSSG after a 5-min pulse

Percentage	C3	C6	C12	C12-Antimycin	C12-DTT	C12-GSSG
Mean	85.2	87.0	87.0	100.0	86.9	94.9
SD	4.3	5.9	4.1	4.9	5.1	5.8
P (c12 vs. others)	0.5	1.0		<0.005	1.0	0.7

(Shown in Figure 4F)
