# Supplementary material for: Control of Precursor Maturation and Disposal Is an Early Regulative Mechanism in the Normal Insulin Production of Pancreatic β-Cells
Source: PLoS One. 2011 Apr 29;6(4):e19446. doi: 10.1371/journal.pone.0019446 (PMC3084858; doi:10.1371/journal.pone.0019446)
Supplement: Table S14 — Proportions of nascent proinsulin monomers and nom-monomers in MIN6 β-cells chased for the indicated times with/without antimycin after a 15-min pulse. (PDF) [file pone.0019446.s017.pdf]

Table S14. Proportions of nascent proinsulin monomers and nom-monomers in MIN6 β-cells chased for the indicated times (minutes) with/without antimycin after a 15-min pulse

Percentage	Proinsulin State	C3	C30	C60	C60A
Mean	Monomers	54	72	83	32
Mean	Non-monomers	46	28	17	68
SD	Monomers	2.7	6.4	9.2	3.6
SD	Non-monomers	2.7	6.4	9.2	3.6
P (Non-monomers, C60 vs.		<0.005	0.04		<0.005

(Shown in Figure 5B)
