# Supplementary material for: Control of Precursor Maturation and Disposal Is an Early Regulative Mechanism in the Normal Insulin Production of Pancreatic β-Cells
Source: PLoS One. 2011 Apr 29;6(4):e19446. doi: 10.1371/journal.pone.0019446 (PMC3084858; doi:10.1371/journal.pone.0019446)
Supplement: Table S15 — Relative levels of nascent proinsulin in MIN6 β-cells chased for the indicated times with/without antimycin after a 15-min pulse. (PDF) [file pone.0019446.s018.pdf]

Table S15. Relative levels of nascent proinsulin in MIN6 β-cells chased for the indicated times (minutes) with/without antimycin after a 15-min pulse

Percentage	C3	C30	C60	C60-Antimycin
Mean	78.3	52.4	24.7	100
SD	4.2	4	3.3	9.7
P (C60-Antimycin vs. Others)	<0.005	<0.005	<0.005	

(Shown in Figure 5C)
