# Supplementary material for: Control of Precursor Maturation and Disposal Is an Early Regulative Mechanism in the Normal Insulin Production of Pancreatic β-Cells
Source: PLoS One. 2011 Apr 29;6(4):e19446. doi: 10.1371/journal.pone.0019446 (PMC3084858; doi:10.1371/journal.pone.0019446)
Supplement: Text S1 — Supplemental Experimental Procedures. (DOC) [file pone.0019446.s019.doc]

**SUPPORTING INFORMATION - TEXT**

# Control of Precursor Maturation and Disposal Is an Early Regulative Mechanism in the Normal Insulin Production of Pancreatic β-Cells

Jie Wang^1*^, Ying Chen^2^, Qingxin Yuan^1§^, Wei Tang^1§^, Xiaoping Zhang^1^, Kwame Osei^1^

^1^Department of Internal Medicine, The Ohio State University, Columbus, Ohio, USA

^2^Departments of Neurobiology and Neurology, The University of Chicago, Chicago, Illinois, USA

*Correspondence to: Dr. Jie Wang, 491 McCampbell Hall, 1581 Dodd Drive, Columbus, OH 43210, USA. E-mail: jie.wang2@osumc.edu *(Phone and Fax numbers: 614-292-0346; 614-292-3084)*

^§^Current address: Division of Endocrinology, The First Affiliated Hospital of Nanjing Medical University, Nanjing, Jiangsu, China

None of the authors of this paper has a financial interest related to this work

**Supplemental Experimental Procedures**

**Ethics statement**

All animal and tissue sample experiments were performed in accordance with the guidelines of the National Institutes of Health and The Ohio State University with procedures (2007A0040 and 2010A0024) approved by the Institutional Animal Care and Use Committee (IACUC) of the university.

**Materials**

We utilized antibodies against mouse C-peptide II (Proteintech Group, Inc., Chicago, IL, USA); glucagon or rat C-peptide II (Linco Research, St. Charles, MO, USA); insulin (Dako North America, Inc., Carpinteria, CA, USA); human C-peptide (Linco Research); tubulin (Sigma-Aldrich, St. Louis, MO, USA); Cytochrome c oxidase subunit 1 (invitrogen); glucose transporter 2 and furin (Santa Cruz Biotechnology, Inc., Santa Cruz, CA, USA); and PDI (Enzo Life Sciences International, Inc., Plymouth Meeting, PA,USA) as well as PC1/3 and PC2 (kindly provided by Dr. Donald F. Steiner, Chicago) and IAPP antibodies (kindly provided by Dr. P. Westermark, Linköping, Sweden). We obtained NEM, DTT, antimycin, GSH, oxidized glutathione, collagenase, bovine serum albumin, Ficoll 400, cycloheximide, tunicamycin, thapsigargin, verapamil, nickel chloride, iodoacetamide, and hydrogen peroxide from Sigma-Aldrich; TNF-α from GenWay Biotech, Inc. (San Diego, CA, USA); methionine and/or cysteine-free Dulbecco's modified eagle medium (DMEM) and Roswell Park Memorial Institute medium (RPMI) 1640 from Invitrogen (Carlsbad, CA, USA); protease inhibitor cocktail from Roche Applied Science (Indianapolis, IN, USA); Immobilon-P^SQ^ membrane from Millipore (Bedford, MA, USA); [^35^S]-methionine/[^35^S]-cysteine and human [^125^I]-proinsulin from PerkinElmer (Waltham, MA, USA) and Linco Research; and Akita heterozygous (*Ins2^+/Akita^)* and C57BL/6J mice from The Jackson Laboratory (Bar Harbor, ME, USA). Cloned *Ins2^+/+^* and *Ins2^+/Akita^*  β-cell lines were kindly provided by Drs. H. Kubota and K. Nagata, Kyoto, Japan; MIN6 β-cells, by Dr. Donald F. Steiner, Chicago, IL, USA; and the human islets, by the Pancreas and Islet Transplant Center at the University of Chicago.

**Islet preparation and cell culture**

Unless specifically stated, we conducted all operations and maintained all materials away from reducing reagents. Islet isolation and islet and cell line culture were described previously (16,18). Briefly, for treatment, we incubated cells in 35-mm dishes in DMEM (25.5 mM glucose) medium supplemented with 10% fetal bovine serum plus (FBS) at 37°C with 5% CO_2_/95% O_2_ until 80 to 90% confluence. We cultured isolated islets overnight in 10% FBS/RPMI 1640 (11 mM glucose) until treatment.

**Pulse-chase**

After 30 minutes’ preincubation in Met-free DMEM for MIN6 β-cells or Met and/or cysteine-free RPMI 1640 medium for islets, mouse islets or MIN6 cells were labeled with ^35^S-Met, and human islets were labeled with ^35^S-Met /^35^S-Cys in the same media. We conducted the various chase tests using pre-balanced complete DMEM/10% FBS media with or without agents. After pulse and/or chase incubations, islets and MIN6 β-cells were quickly washed twice with PBS containing 20 mM NEM and immediately lysed in the immunoprecipitation (IP) buffer and/or frozen at -80 ^o^C.

**Protein extraction buffers and sample preparations for immunoblotting and immunoprecipitation**

We extracted protein using the following buffers and solutions: radioimmunoprecipitation assay (RIPA) buffer, a cocktail of 50 mM Tris-HCl, pH 7.4, 1% Triton X-100, 1% sodium deoxycholate, 0.1% sodium dodecyl sulfate (SDS), 150 mM NaCl, 5 mM ethylenediaminetetra-acetic acid (EDTA), 20 mM NEM, and protease inhibitor; tricine gel sample buffer, comprising 100 mM Tris, pH 6.8, 1% SDS, 20% glycerol, 20 mM NEM, and 0.02% Coomassie Blue; and IP buffer, a cocktail of 50 uM Tris⋅HCl, pH7.4, 100 mM NaCl, 2.5 mg/mL BSA, 1% Triton X-100, 20 mM NEM, and protease inhibitor.

We harvested proteins using several sample preparation procedures (SPP). SPP-A was used to prepare the samples used in Figure 1A. Briefly, cultured islets were quickly washed twice with PBS/ 20 mM NEM and immediately lysed in RIPA buffer. The lysates were mixed well by vortex for 30 seconds 3 times at room temperature and then placed on ice for 20 minutes. After insoluble materials were spun down at 14,000 g at 4 ^o^C for 10 minutes, we collected soluble protein extracts to measure protein concentration (Bio-Rad) and stored them at -20°C until use. We used SPP-B to prepare the samples in Figures S1B and Figure 6. Briefly, we solubilized cells or the acid-alcohol insoluble precipitates in the tricine gel sample buffer and sonicated the lysate for 30 seconds on ice 6 times. After spinning down insoluble materials at 14,000 g for 10 minutes at 4 ^o^C, we transferred the soluble protein extracts into new tubes for quantitative analysis of protein concentration and stored them at -20°C until use. SPP-C was applied to prepare the samples in all IP tests in this article (Figs. 2-5). Briefly, islets and MIN6 β-cells were lysed in the IP buffer and sonicated for 30 seconds on ice 4 times***.*** We then centrifuged the supernatants at 14,000 × g for 5 min at 4 ^o^C, incubated them with antibodies at 4°C for 12 hours and then Protein A-Sepharose™ (Bio-Rad) for another 6 hours with gentle rotation. We washed the immunoprecipitates 3 times with the IP buffer and then suspended them in a tricine gel sample buffer for use. In the experiment in Figures S2, we extracted unlabeled proteins of *Ins2^+/+^* and *Ins2^+/Akita^* islets in the IP buffer, in which human ^125^I-proinsulin marker (Linco) was already added, and then subjected them to the same IP procedure.

**SDS-PAGE**

We boiled protein samples for 10 minutes in tricine gel sample buffer with and without 100 mM of DTT for SDS-PAGE. Tricine-SDS-PAGE gels without urea were applied for all other tests in this article except the one with urea (Fig. 3A) and the 7.5% Laemmli SDS-PAGE (Fig. 4D). Gel concentrations (%T, % C; T denotes the total percentage concentration of both acrylamide and bisacrylamide; C denotes the percentage concentration of the bisacrylamide relative to the total concentration) are described in figure legends.

**Immunoblotting and radioautography**

We performed immunoblotting following standard procedures using antibodies against C-peptide or tubulin. For autoradiography, we dried membrane and fixed gels with labeled materials for exposure on x-films.

**Quantitative analysis of immunoreactivity and radioactivity**

To quantify the density and radioactivity of protein bands (area and/or gel slices), we used National Institutes of Health (NIH) Image J software and/or liquid scintillation counter and gamma counter (Beckman Coulter, Inc., Brea, CA, USA*).*

**Methods of calculating the proportions of proinsulin or control protein monomers and non-monomers**

In general, the amount of protein monomer(s) in a reduced SDS-PAGE/membrane (by adding DTT) is accepted as the total level of protein in loaded samples. Resolution of equal amounts of protein in individual samples by SDS-PAGE under non-reduced and reduced conditions permits calculation of the fractions of protein monomers and non-monomers (referred to as DTT-sensitive forms) in individual samples. The resolution is possible because conversion of protein from its DTT-sensitive non-monomer states to monomers by the effect of DTT yields different protein monomer levels between the two conditions. We calculated the proportions of proinsulin or control protein monomers and non-monomers shown in the following sections by the formulas: monomers (%) = (monomer level under non-reduced condition) X 100% / (monomer level under reduced condition); non-monomers (%) = (monomer level under reduced condition - monomer level under non-reduced condition) X 100% / (monomer level under reduced condition); or non-monomers (%) = 100% - monomers (%).

**Data analysis**

Data are presented as the mean ± standard deviation (SD; n = 3 to 6). We assessed statistical significance (**P* < 0.05; ***P* < 0.005) by Student’s t-test (2-tailed) or analysis of variance (ANOVA) if appropriate.
